# MiR674 inhibits the neuraminidase-stimulated immune response on dendritic cells via down-regulated Mbnl3

**DOI:** 10.18632/oncotarget.9832

**Published:** 2016-06-06

**Authors:** Jian Lin, Ya T. Chen, Jing Xia, Qian Yang

**Affiliations:** ^1^ Life Science College, Nanjing Agricultural University, Weigang, Nanjing, Jiangsu, PR China; ^2^ College of Veterinary Medicine, Nanjing Agricultural University, Weigang, Jiangsu, PR China

**Keywords:** dendritic cells, neuraminidase, miR-674, immune regulation, virus replication, Immunology and Microbiology Section, Immune response, Immunity

## Abstract

Neuraminidase (NA), a structural protein of the H9N2 avian influenza virus (H9N2 AIV), can facilitate viral invasion of the upper airway by cleaving the sialic acid moieties on mucin. Dendritic cells (DCs) are major antigen-presenting cells whose immune functions, such as presenting antigens and activating lymphocytes, can be regulated by microRNAs. Here, we studied the molecular mechanism of miRNA-induced repression of immune responses in mouse DCs. First, we screened for and verified the miRNAs induced by NA. Then, we showed that, consistent with the H9N2 virus treatment, the viral NA up-regulated the expression of miR-155, miR-674, and miR-499 in DCs; however, unlike H9N2 virus treatment, the presence of NA was associated with reduced expression of miR-181b1. Our results suggest that NA significantly increased DC surface markers CD80 and MHCII and enhanced the ability of activating lymphocytes and secreting cytokines compared with HA, NP and M2. Meanwhile, we found that miR-674 and miR-155 over-expression increased all surface markers of DC. Nevertheless, by inhibiting the expression of miR-674 and miR-155, NA lost the ability to promote DC maturation. Furthermore, we predicted and demonstrated that *Pgm2l1, Aldh18a1, Camk1d*, and *Mbnl3* were the target genes of miR-674. Among them, *Mbnl3* interference strongly blocked the mature DCs. Collectively, our data shed new light on the roles of and mechanisms involved in the repression of DCs by miRNAs, which may contribute to efforts to develop a prophylaxis for the influenza virus.

## INTRODUCTION

Frequent outbreaks of reassortant H7N9 avian influenza virus (AIV) caused great threatened to the poultry industry and the public health [1]. Sequencing analyses revealed that six internal genes of H7N9 subtype shared the highest similarity with H9N2 subtype viruses that have circulated in poultry [2]. H9N2 avian influenza virus (AIV) has caused multiple disease pandemics due to its high genetic variability and high rate of recombination with other influenza virus subtypes. Neuraminidase (NA) was an important target for host neutralizing antibodies. Point mutations in the antigenic domains of the NA protein were considered as a way to escape host immune system for viruses [3]. Eckard suggested that NA may be another piece of the influenza vaccine, while European Pharmacopeia suggest to maximize vaccine NA content to induce protective immunity against influenza infection at the same time [4]. So the big question now is how virus protein NA triggered the immune response.

Since dendritic cells (DCs), professional antigen presenting cells, acted as sentinels for monitoring pathogenic microbes, allergens, and pollutants [5]. It is time to propose that if and how NA segment induced the immune activation of DCs. As we known, the uptake of AIV is necessary for DCs triggering innate and subsequent adaptive immune responses to against virus invasion and replication [6]. Studies found that AIV infection can regulate the maturation, the antigen presenting and the cytokines secretion of DCs [7]. Consider NA is a surface glycoprotein of influenza viruses and can induce NA-directed antibody responses that are independently predictive of protection [8]. We need further study how DCs recognized NA and triggered the immune defending for achieving the goals of controlling and eliminating influenza virus effectively.

Vaccination with inactivated vaccine has been a main measurement to prevent avian influenza virus for a long time [9, 10]. Given the limitations of the current prevention or treatment of acute influenza, novel therapies are needed. On one hand, RNA interference (RNAi) through microRNAs (miRNAs) is an emerging technology that can suppress virus replication *in vitro* and *in vivo* [11]. For example, temporal and specific host miRNA let-7c has been demonstrated inhibit M1 protein expression of the H1N1 influenza virus in infected human lung epithelial cells [12]. On the other hand, miRNAs affect the development of DCs and their ability to present antigens as well as secret cytokines [13], which may block the infection of AIV. For example, miR451 regulates a subset of pro-inflammatory cytokine to combat with influenza virus in mice DCs [14]. In addition, there are now solid data to suggest a protective role for NA immunity and to prove that contribution of antibody production against neuraminidase to the protection afforded by influenza vaccines [4, 15]. The purpose of our study was to figure out how miRNAs regulate the immune response of DCs stimulated by virus protein NA.

## RESULTS

### Distinctive alteration of miRNAs in response to viral fragment stimulation

**Figure 1:**
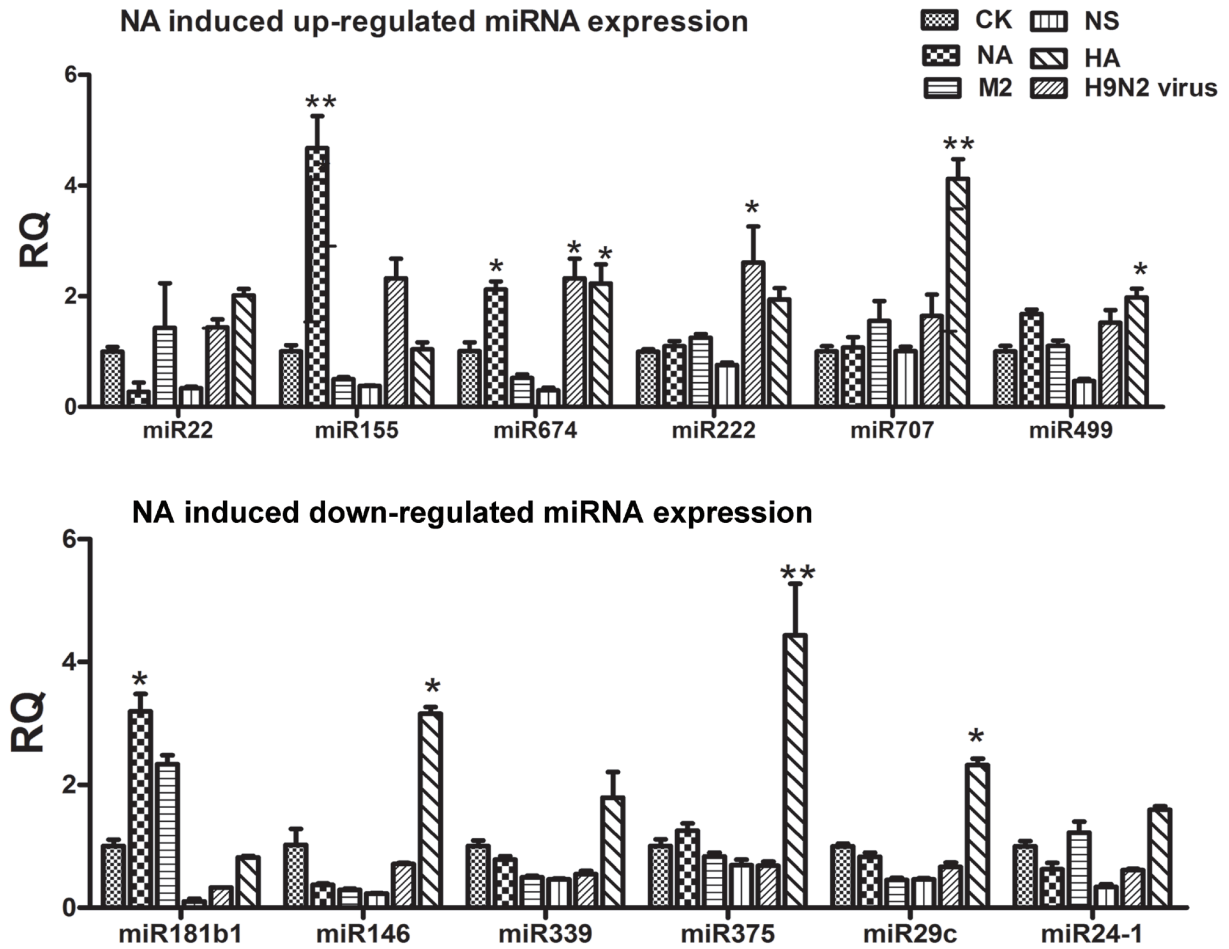
Results of the qPCR analysis of select miRNAs following stimulation by NA, HA, M2, or NS **A.** The expression levels of up-regulated miRNAs stimulated by NA, HA, M2, and NS. **B.** The expression levels of down-regulated miRNAs stimulated by NA, HA, M2, and NS.

Previously, we studied the influence of H9N2 AIV infection on global RNA expression in mice. We found that nine miRNAs were significantly up-regulated by viral infection and that eight were down-regulated (Supplement 1). To study how H9N2 might control miRNA expression, we studied four segments of the H9N2 virus that were unrelated to replication: neuraminidase (NA), hemagglutinin (HA), matrix protein 2 (M2), and non-structural protein (NS). These genes were cloned into the pcDNA3.1 vector (Supplement 2) and transfected into bone marrow-derived DCs (BMDCs). The expression of select miRNAs was examined by quantitative PCR (qPCR). We found that the NA segment significantly increased the expression of miR-155 and miR-674, whereas the HA segment significantly up-regulated the expression of miR-707, miR-674, and miR-499 (Figure 1A). For those miRNAs down-regulated by H9N2, the NA segment greatly increased the expression of miR-181b1; however, unlike the H9N2 virus treatment, the HA treatment significantly rose the expression of miR146, miR375, and miR-29c, (Figure 1B).

### Activation of mouse BMDCs by NA

**Figure 2:**
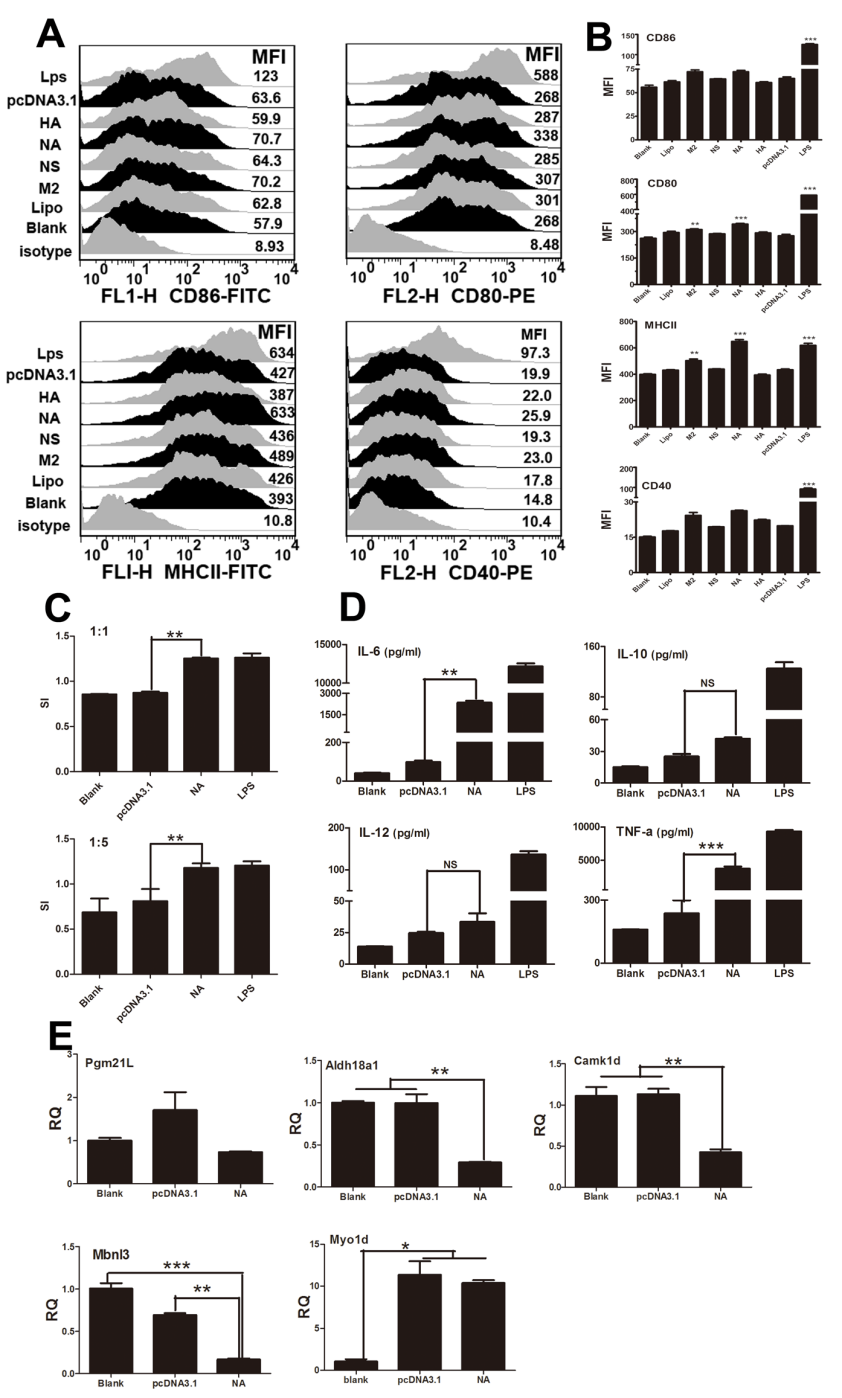
Immune activation of BMDCs stimulated by NA **A.** Flow cytometric analysis of the phenotypic alterations in DCs stimulated with NA, HA, M2, or NS (i.e. the expressions of CD40, CD80/86, and MHCII on BMDCs stimulated with NA, HA, M2, or NS). Positive control, 1 μg/ml LPS. **B.** The MFI of CD40, CD80/86, and MHCII. **C.** NA-stimulated BMDCs stimulated the proliferation of naive T cells in mixed-lymphocyte reactions (MLR). The stimulator cells were BMDCs stimulated with or without NA, pcDNA3.1, or LPS at 37°C for 24 h. All experiments were performed at least in triplicate. Significant differences between the treated and control groups are expressed as *P < 0.05 or **P < 0.01. **D.** Cytokine release from NA-stimulated BMDCs was measured by enzyme-linked immunosorbent assays (ELISAs). Data for IL-6, IL-10, IL-12, and TNF-α are shown as the mean ± standard deviation (SD) of three samples. **P < 0.01, *P < 0.05 compared with the LPS-only group. All results are representative of three independent experiments. **E.** Results of the qPCR analysis of select target genes (*Myo1d, Pgm2l1, Aldh18a1, Camk1d*, and *Mbnl3*) following stimulation by NA.

Next, we investigated how the viral segments affect mouse BMDC functions. There are three standards for evaluating the immune function of BMDCs: phenotypic alterations, the ability to activate T lymphocytes, and the ability to secrete cytokines [16]. We first examined the phenotypic changes of DCs after stimulation with NA, HA, M2, and NS. Fluorescence-activated cell sorting (FACS) suggested that viral segments NA and M2 significantly enhanced the percentages and mean fluorescent intensity (MFI) of MHCII, as well as of the co-stimulatory molecule CD80 (Figure 2A and 2B). The HA segment up-regulated only CD40, whereas NS had no effect (Figure 2A and 2B). Next, we assessed the ability of DCs to activate lymphocytes and secrete cytokines. As shown in Figure 2C and 2D, DCs treated with NA displayed enhanced stimulation at a ratio of 1:1 or 1:5, and higher levels of interleukin (IL)-6 and tumour necrosis factor (TNF)-α were observed compared with the pcDNA3.1-transfected controls (*P < 0.05*). CD40, CD80/CD86, and MHCII are characteristic surface markers for fully matured DCs. Additionally, we found that the mRNA expression of Pgm21l, Aldh18a1, Camk1d, and Mbnl3, targeted to miR674, decreased significantly when treated with NA (Figure 2E). Myo1d, a target gene of miR155, was greatly down-regulated in the NA group compared with the pcDNA3.1 group (Figure 2E).

### Immune function of miR-674 and miR-155 in regulating mice BMDCs

**Figure 3:**
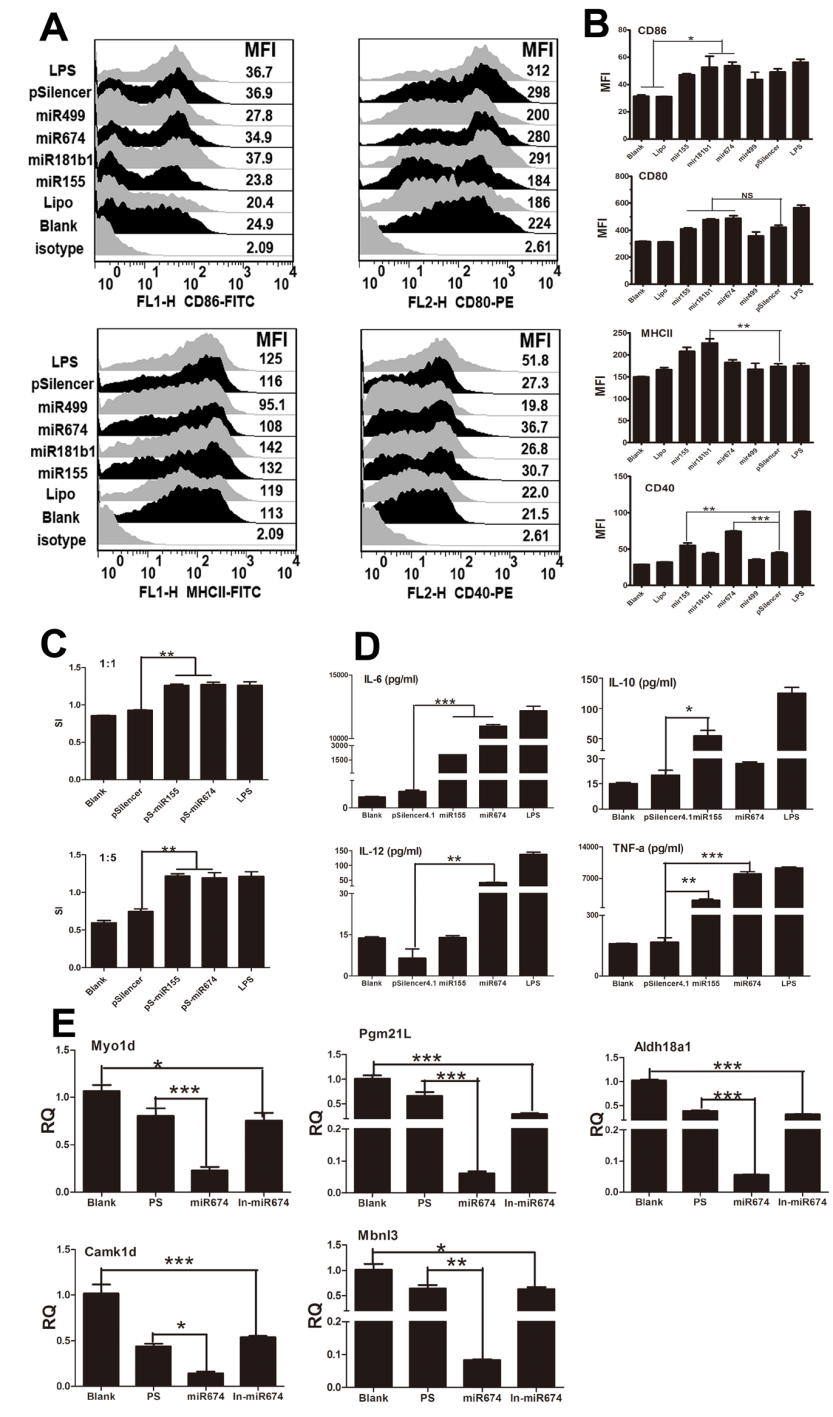
Immune function of BMDCs stimulated by miRNAs **A.** Flow cytometric analysis of the phenotypic alterations in DCs stimulated by miR155, miR499, miR375, miR674, or miR181b1 (i.e. the expressions of CD40, CD80/86, and MHCII on BMDCs stimulated by miRNAs). Positive control, 1 μg/ml LPS. **B.** The MFI of CD40, CD80/86, and MHCII. **C.** miR-155- or miR-67-stimulated BMDCs stimulated the proliferation of naive T cells in MLR. The stimulator cells were BMDCs stimulated with or without miR29c, pSilencer, or LPS at 37°C for 24 h. All experiments were performed at least in triplicate. Significant differences between the treated and control groups are expressed as *P < 0.05 or **P < 0.01. **D.** Cytokine release from BMDCs stimulated by miR-155 or miR-674 measured by ELISA. Data for IL-6, IL-10, IL-12, and TNF-α are shown as the mean ± SD of three samples. **P < 0.01, *P < 0.05 compared with the LPS-only group. All results are representative of three independent experiments. **E.** Results of the qPCR analysis of select target genes *Myo1d, Pgm2l1, Aldh18a1, Camk1d*, and *Mbnl3*) following stimulation by miR155 or miR674.

Recent studies have shown that miRNAs regulate the immune responses of BMDCs [17]. Given that the H9N2 virus and NA segment significantly up-regulated the expression of miR-155 and miR-674 (Figure 1A), we examined the functions of these miRNAs in BMDCs. miRNA over-expression vectors were constructed and validated, as shown in Supplement 3. FACS results indicated that miR-674 and miR-155 significantly increased the percentage and MFI of CD40-positive cells (Figure 3A and 3B) compared with the pSilencer group. Interestingly, miR-181b1 increased the percentage and MFI of MHCII-positive cells. Moreover, overabundance of miR-155 and miR-674 promoted lymphocyte proliferation at a ratio of 1:1 and 1:5 respectively (Figure 3C). Furthermore, we found that miR-674 increased the expression of IL6, IL-12, and TNF-α, whereas miR-155 induced IL-6, IL-10, and TNF-α production (Figure 3D). Additionally, we found that the mRNA expression of Myo1d, Pgm21l, Aldh18a1, Camk1d, and Mbnl3, targeted to miR155 or miR674, were significantly decreased in miR155 and miR674 over-expression groups compared with the pSilencer4.1 control group (Figure 3E).

### Inhibition of endogenous miRNA function blocked NA-induced phenotypic alterations in BMDCs

**Figure 4:**
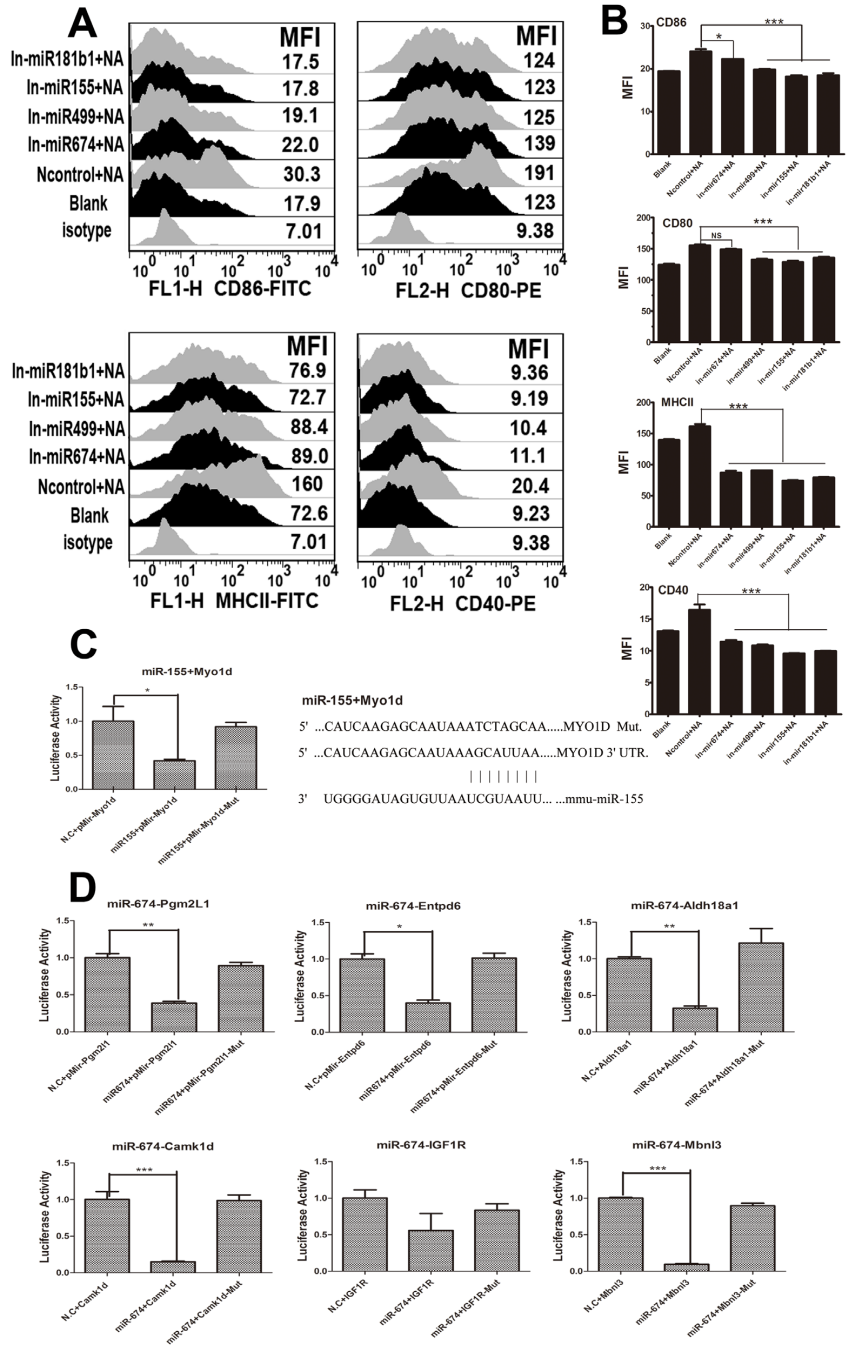
Results of miRNA inhibitor treatment and dual luciferase reporter assays for miR155, miR674, and their target genes **A.** The expressions of CD40, CD80/86, and MHCII on BMDCs when miR-155, miR-674, miR-499, and miR-181b1were inhibited (100 μg/ml inhibitor for each miRNA). **B.** The MFI data for CD40, CD80/86, and MHCII. **C.** The map position of gene Myo1d and its mutated site targeted to miR155, and the dual luciferase assay results for miR-155 and its target genes (level of significance: *P < 0.05* or *P < 0.01*). **D.** Dual luciferase assay results for miR-674 and its target genes (level significance: *P < 0.05* or *P < 0.01*).

Previous studies have demonstrated that NA and miR-674 or miR-155 have similar effects on BMDCs. Thus, we investigated whether the NA-induced changes in DCs are mediated by miRNAs. To test this hypothesis, miRNA inhibitors were designed and added to DCs to repress endogenous miRNAs prior to NA transfection. FACS revealed that the inhibition of endogenous miR-674 or miR-155 decreased the expression of co-stimulatory molecules (CD80/CD86 and CD40) and MHCII, which was induced by NA. Moreover, NA did not increase the MFI of CD40- and MHCII-positive cells when miR-499 or miR-181b1was inhibited (P < 0.05) (Figure 4A and 4B).

### MiR-674 and miR-155 target prediction and validation

miRNAs exert their functions by causing translational repression or mRNA degradation. To study the mechanism of action by miR-155 and miR-674, we used Miranda and Targetscan to predict miR-155 and miR674 target genes, considering only the overlap of both algorithms, and further matched the prediction based on our previous microarray data. Finally, we selected *Myod1* targeted to miR-155 and *Pgm2l1, Entpd6, Aldh18a1, Camk1d, Igf1r, or Mbnl3* targeted to miR-674 for further testing. Luciferase reporter assays showed that *Pgm2l1, Aldh18a1, Camk1d,* and *Mbnl3* groups significantly decreased luciferase activity (P < 0.05), whereas *Entpd6* and *Igf1r* groups non-significantly reduced luciferase activity (Figure 4D). Similar results were observed in the *Myo1d* group; luciferase activity decreased markedly once we added miR-155, which did not occur in the *Myo1d*-muted group (Figure 4C).

### Silenced endogenous target gene blocked NA-induced phenotypic alterations in BMDCs

**Figure 5:**
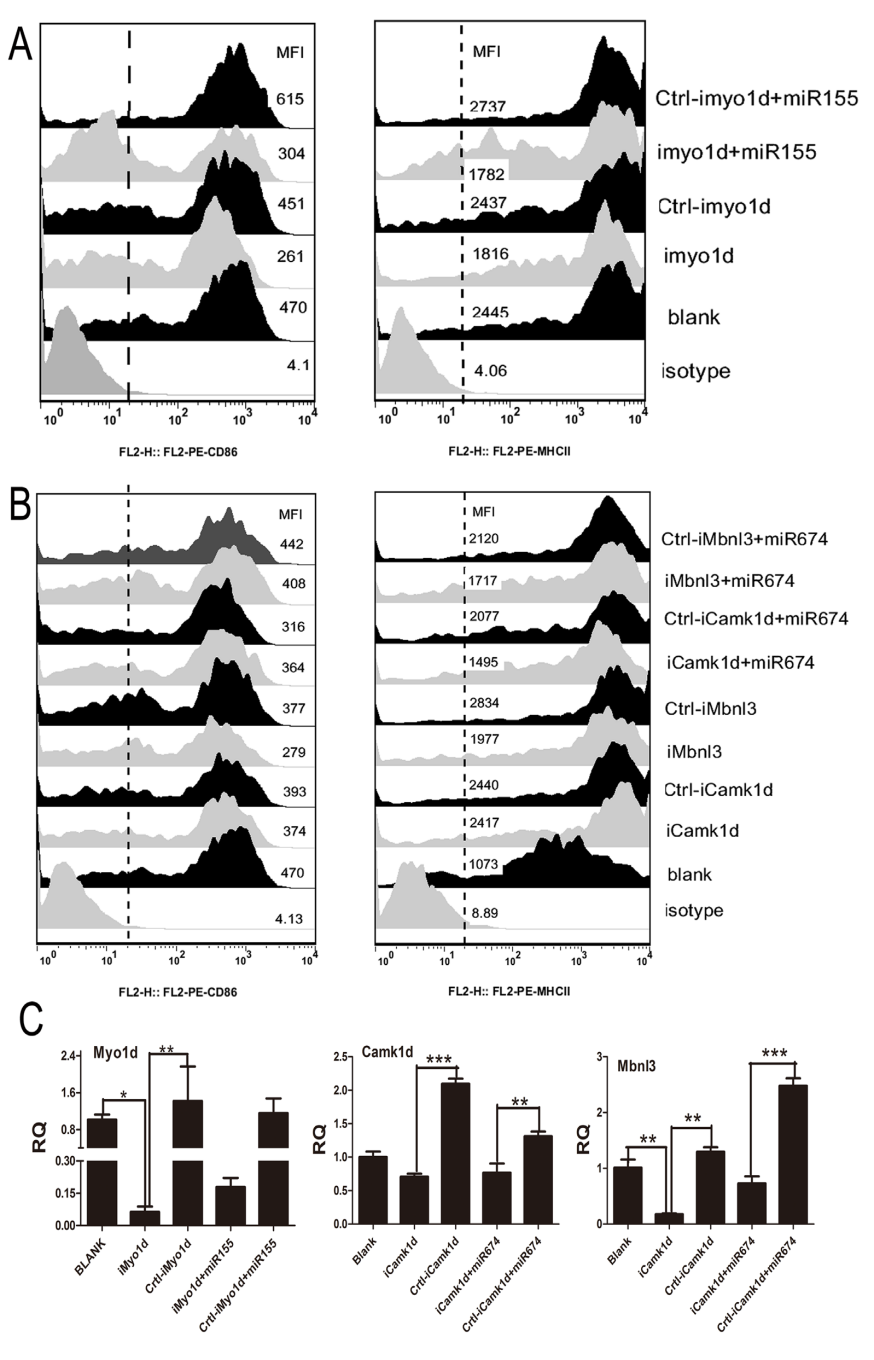
Results of target gene silencing treatment on DC phenotypic alterations **A.** The expression of CD86 and MHCII on BMDCs when the target gene (Myo1d) of miR-155 was inhibited (DCs were divided into Isotype, Blank, iMyo1d, Crtl-iMo1d, iMyo1d/miR155, and Crtl-Myo1d/miR155). **B.** The expression of CD86 and MHCII on BMDCs when the target genes (Camk1d and Mbnl3) of miR-674 were inhibited (DCs were divided into Isotype, Blank, iCamk1d, Crtl-iCamk1d, iMbnl3, Crtl-iMbnl3, iCamk1d/ miR674, Crtl-iCamk1d/ miR674, iMbnl3/ miR674, and Crtl-iMbnl3/ miR674). **C.** qPCR analysis of the RNA interference results of Myo1d, Camk1d, and Mbnl3 in the above treatments.

Our study demonstrated that *Myo1d, Camk1d,* and *Mbnl3* were the target gene of miR-155 and miR-674. To determine whether the knockdown of *Myo1d*, *Camk1d,* and *Mbnl3* had any gross effect on the immune function of BMDCs, we assessed the phenotypic alteration of DCs when these genes were knocked down or over-expressed. On one hand, qPCR results revealed that the mRNA levels of all three genes were reduced after siRNAs lentivirus vector transfection (Figure 5C). Of these siRNAs, the greatest decrease in *Myo1d* mRNA levels was achieved using siMyo1d-1, whereas siCamk1d-9 and siMbnl3-15 significantly reduced their mRNA expression levels compared with the negative control. FACS results consistently suggested that BMDCs lose the ability to raise their surface marker CD86 and MHCII when *Mbnl3* or *Myo1d* genes were silenced (Figure 5A and 5B). Furthermore, the inhibitory effect of silencing *Mbnl3* or *Myo1d* genes did not recover any further even when their mirror miRNAs were added to DCs (Figure 5A and 5B). These data strongly suggested that *Mbnl3* or *Myo1d* may be the next target for combating influenza virus. On the other hand, FACS result suggested that the over-expression of *Mbnl3* hugely increased the surface marker CD86 and MHCII, whilst the over expressed *Mbnl3* had no effect on CD40 and CD80 (Supplement 9).

### Effects of NA, miR-674, and miR-155 on signalling pathways

Previously, we demonstrated that H9N2 AIV activates interferon (IFN) regulatory factor (IRF)-7 and TNF receptor-associated factor (TRAF)-6. Thus, we continued to explore the signalling pathways that may be activated by NA and miR155 or miR674. As we known, mitogen-activated protein kinase (MAPK) pathways exist in all eukaryotes and control a wide range of cellular processes, such as proliferation, differentiation, and survival. In this study, we found that NA segment induced the activity of IkBa and P38 signal pathway by phosphorylating it. Whilst, NA stimulation has no effect or decreased the JNK and ERK signal pathways (Supplement 7). Moreover, we found that miR-155 overabundance activated both IkBa and P38 signalling pathways, while miR-674 over-expression group just activated the P38 signal pathway(Supplement 7). Furthermore, we found that NA stimulation significantly down-regulated the expression of IRF-3, TRAF-3, and-6. Additionally, the expression of IRF-3 and TRAF-3 were slightly decreased in miR-674 group, compared with blank group. In terms of the miR-155 over-expression group, the levels of IRF-7 and TRAF3/6 increased slightly compared with the decreased IRF-3 expression (Supplement 6). These results not only indicate that the activation of P38 pathway may involved in the regulation of the DC function when NA or miR-674 stimulation, but also suggested that IRF-3 might be involved in the regulating of NA-stimulated DC immune function.

### Effects of miR674 or miR155 inhibition or over-expression on virus replication

**Figure 6:**
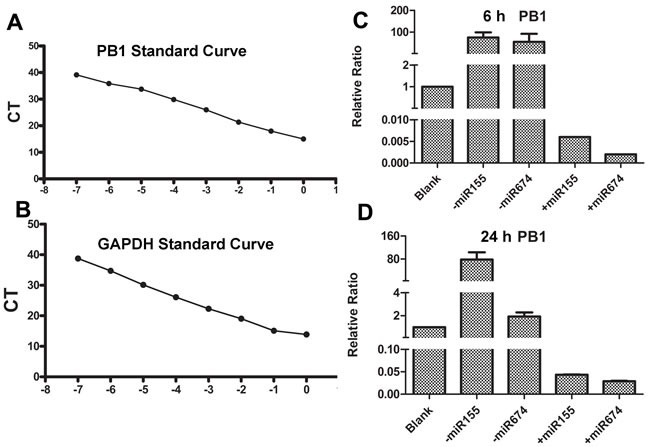
Detection of viral replication in DCs when miR155 and miR674 were inhibited or over-expressed **A.** Standard curve for the PB1 gene from H9N2 AIV. **B.** Standard curve for the mouse GAPDH gene. **C.** Replication of H9N2 AIV following pre-treatment of DCs with miRNAs for 6 h. **D.** Replication of H9N2 AIV following pre-treatment of DCs with miRNAs for 24 h.

Considering that miR674 and miR155 significantly affected NA-induced DC function, we evaluated whether miR674 or miR155 could influence the replication of H9N2 AIV. We generated standard curves for PB1 and GAPDH (Figure 6A and 6B) and then conducted absolute qPCR. Our results showed that the inhibition of miR674 or miR155 significantly up-regulated viral replication at 6 and 24 h, whereas the over-expression of miR674 or miR155 repressed viral replication (Figure 6C and 6D).

## DISCUSSION

The interactions between cellular factors, such as miRNAs and H9N2 AIV, are important for AIV infection and replication. In this report, we found that the NA segment of H9N2 AIV modulated the immune function of DCs by adjusting miR-155 and miR-674 expression. NA, an envelope glycoprotein on the surface of the influenza virus, catalyses the cleavage of sialic acids on glycoproteins from virus-infected cells and enables virus release [18, 19]. Studies have demonstrated that low NA enzyme activity renders virus release from infected cells inefficient and leaves progeny viruses gathered on the cell surface [19, 20]. DCs represent a central element in the generation and maintenance of immune responses [21]. Our study suggested that the immune functions of DCs, including phenotypic alteration, T lymphocyte activation, and cytokine secretion, were greatly stimulated by NA. Additionally, we identified four cellular miRNAs (miR-155, miR-674, miR-499, and miR-181b1) that were significantly up-regulated by NA. Among them, miR-155 and miR-674 were shown to support the ability of DCs to activate lymphocytes.

MiR-155 shapes the balance between tolerance and immunity, which plays a critical role in immunity and viral infections [22]. miR-155 is involved in fine-tuning the regulation of lymphocyte subsets, such as B cells, CD8^+^, and CD4^+^ T cells including T helper type 1 (Th1), Th2, Th17, and regulatory T cells [23]. Considering that the level of miR-155 is correlated with immune function, we evaluated the potential role of miR-155 in regulating DC immune responses. The current results suggest that increased NA may enhance DC immune response by increasing the levels of miR-155. Our research suggests that increased miR-155 enhances the DC immune responses induced by NA by increasing their ability to activate lymphocytes.

We found that over-expressed NA up-regulated the expression of CD80 and MHCII in cultured BMDCs, whereas the inhibition of endogenous miR-155 had the opposite effect. MiR-155 also modestly raised the expression of surface marker CD40; this effect was reversed by inhibiting endogenous miR-155. CD40 is a member of the TNF receptor family, which promotes the development of T cell immune responses by binding to CD40L [24]. Thus, miR-155 may enhance DC-T cell interactions by up-regulating DC surface maturation molecules. Moreover, increased NA or miR-155 also affected the ability of DCs to activate lymphocytes and secrete inflammatory cytokines. Our data revealed that NA and miR155 increased the expression of the pro-inflammatory cytokines IL-6, IL-10, and TNF-α.

Compared with research on miR-155, studies on miR-674 are scarce. The current study is the first to focus on the role of miR-674 in evoking the immune activation of BMDCs. Functional experiments demonstrated that miR-674 significantly increased the percentage ratio and MFI of CD40- and CD80-positive cells in cultured BMDCs and that inhibition of the endogenous miR-674 reversed the effects. Moreover, miR-674 enhanced the ability of DCs to activate lymphocyte cells and secret cytokines, two additional standards for evaluating DC function [16]. Additionally, NA stimulation also promoted the above two functions. For cytokines, our data suggested that miR-674 increased the expression of IL-6, IL-12, and TNF-α but had no effect on anti-inflammatory cytokine IL-10. DCs promote the Th1 response *via* IL-12 [25]. Accumulated IL-12 and enhanced proliferation of lymphocyte cells demonstrated that DCs matured and became activated when stimulated by miR-674.

Host defence against viral invasion involves activating the innate immune system with IFN-α [26]. The transcription of IFN-α is controlled primarily by members of the IFN regulatory factor family (specifically, IRF-3 and IRF-7) [27]. Viral infection results in the activation of IRF-3 and the low-level secretion of IFN-α on non-plasmacytoid DCs [28]. Previous research has demonstrated that virus protein NS1 activates IRF-3-mediated IFN-α expression [29, 30]. Nevertheless, our data showed that IRF-3 was down-regulated in the NA and miR-674 treated group, indicating a repression of IFN-α production. This finding is consistent with our hypothesis that NA impacts DC functions by up-regulating miR-155 and miR-674.

Most genes targeted by miR-674 are involved in immune and inflammatory responses. Additionally, based on the identified genes, *Pgm2l1, Entpd6, Aldh18a1, Camk1d, Igf1r,* and *Mbnl3* were especially distinctive in the gene interaction network. Our results showed that miR-674 repressed the expression of *Pgm2l1, Aldh18a1, Camk1d,* and *Mbnl3*. Of these target genes, the silence of *Mbnl3* could decrease the surface marker of CD86 and MHCII, which might represent a mechanism for regulating DCs. Signal pathway molecules and adaptor proteins are identified as strong miRNA targets. For example, TAK1-binding protein, targeted to miR155, is elevated to activate the p38 pathway [31]. TRAF6, a target of miR-146, is important for NF-kB activation [32]. Our data demonstrated that both NA and miR-155- or miR-674-treated groups activated p38 pathways, which might be contributed to the regulation of IFN-α by decreasing IRF-3.

**Figure 7:**
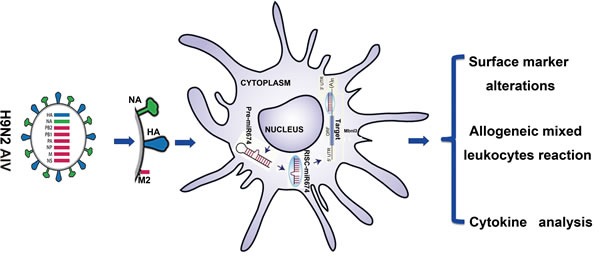
Summary of the research strategy

In summary, our results suggest that cellular miRNAs are important factors in regulating host DC functioning related to defending against viral infection and provide a deeper understanding of the mechanisms underlying the host defence system. We demonstrated a previously unidentified role for miR674 in the activation of immune responses. We suggest that miR674 mediates the NA-induced immune responses related to DCs by targeting and repressing *Mbnl3* gene (Figure 7). Thus, miR-674 may be a new immune reinforcing agent that can be used in activating the DC function involved in defending against H9N2 AIV.

## MATERIALS AND METHODS

### Virus and animals

Influenza A virus (A/duck/Nanjing/01/1999(H9N2)) was provided by the Institute of Animal Husbandry and Veterinary Medicine, Jiangsu Academy of Agricultural Science (Nan Jing, China). Allantoic fluid was concentrated 10-fold (10^9^ egg infectious doses 50 (EID50) / 0.1 ml) and purified on a discontinuous sucrose density gradient as described [33]. SPF C57BL/6 and BALB/c mice were obtained from Comparative Medical Center of Yang Zhou University.

### Ethics statement

This study was approved by the Ethical Committee of Animal Experiments of the College of Veterinary Medicine, Nanjing Agricultural University. All animal care and use were conducted in strict accordance with the Animal Research Committee guidelines of the College of Veterinary Medicine, Nanjing Agricultural University.

### Plasmids and cell culture

Four segments unrelated to viral replication (NA, HA, M2 and NS) were amplified from the H9N2 virus and cloned into pcDNA3.1 (Invitrogen). Primers were listed in Table 1. MiRNAs (miR-155, miR-674, miR-499 and miR-181b1) were amplified and cloned into pSilencer4.1 (Invitrogen), whose primers were listed in Table 2. The 3′-UTRs of *Myo1d* mRNAs target to miR-155 and the 3′-UTRs of *Pgm2l1, Aldh18a1, Entpd6, Camk1d, Igf1r* and *Mbnl3* mRNAs, harboring the predicted miR-674 binding sequences, were amplified and cloned into pMIR-Report luciferase vector (Ambion, TX) with primers listed in Table 3. The shRNA target to *Myo1d, Camk1d* and *Mbnl3* gene were designed and purchased from Invitrogen. Primers are list in Table 4. The artificial synthesis shRNA was then annealed and cloned into the BamHI and EcoRI sites of pLVX-shRNA1 using compatible restriction sites flanking the oligo siRNA target sequence. The expressing vector pLVX-shRNA1 expression vector and lentivirus transfection packaging kit (Lenti-X Packaging System) were purchased from Clontech (Mountain View, CA, USA).

**Table 1:** Primers used in amplified NA, M2, NS and HA

Gene	Sequence	Products
**NA Sence**	G*GGTACC*ATGAATCCAAATCAGAAG	**1418 bp**
**NA Anti-sence**	G*CTCGAG*TTATATAGGCATGAAGTTG	
**M2 Sence**	G*GGTACC*ATGAGTCTTCTAACCGAG	**773 bp**
**M2 Anti-sence**	G*CTCGAG*TCACTTGAATCGCTGC	
**NS Sence**	G*GGTACC*ATGGATTCCAACACTG	**707 bp**
**NS Anti-sence**	G*CTCGAG*TTAAATAAGCTGAAACGAG	
**HA Sence**	GGGTACCATGGAAGTAGTATCACTAAT	
**HA Anti-sence**	GCTCGAGTTATATACAAATGTTGCATC	**1683 bp**

**Table 2:** Primers used in amplified miR155, miR674, miR181b1 and miR499

Gene	Sequence	Products
**MiR155 Sence**	GCG*GGATCC*TATTTCTTTTTCTCTTTG	**485 bp**
**MiR155 Anti-sence**	GCG*AAGCTT*ACTGCTGATCTATCTTTTA	
**MiR181b1 Sence**	GCG*GGATCC*ATTCAAATAAAAACCATC	**504 bp**
**MiR181b1 Anti-sence**	GCG*AAGCTT*TTAGTGACTTCCTCACAG	
**MiR674 Sence**	GCG*GGATCC*TTAACTCAACTACCCAGGT	**530 bp**
**MiR674 Anti-sence**	GCG*AAGCTT*ACAAAAATCCCCAAACAC	
**MiR499 Sence**	G*GGATCC*CGCAAGAAAGCAGCATC	**503 bp**
**MiR499 Anti-sence**	G*AAGCTT*CACCCCAAACACCACCT	

**Table 3:** Primers used in amplified miRNAs target genes

Gene	Sequence	Products
**MiR155 targets**		
**Myo1d Sence**	ATA*GAGCTC*CAGCACCTGGTCTCCTAC	**630 bp**
**Myo1d Anti-sence**	GC*AAGCTT*AAGATTTAATGCTTTATTGCTC	
**MiR674 targets**		
**Pgm2l1 Sence**	ATA*GAGCTC*GCATGTACGGGACATAAC	**530 bp**
**Pgm2l1 Anti-sence**	C*AAGCTT*TGCCACTGGAACACTTTC	
**Entpd6 Sence**	ATA*GAGCTC*TGGAAGTGACACCATCCT	**880 bp**
**Entpd6 Anti-sence**	GC*AAGCTT*AAACCCTTGTAAACCTTTAT	
**Aldh18a1 Sence**	ATA*GAGCTC*TCTTGGAAGGGTCTGTCTT	**720 bp**
**Aldh18a1 Anti-sence**	C*AAGCTT*CTGGAGGTCGAGAATAGG	
**Camk1d Sence**	ATA*GAGCTC*CCTGCATAGGACTGGAAG	**548 bp**
**Camk1d Anti-sence**	GC*AAGCTT*CCTCTGGAAGAAGGGACT	
**Igf1r Sence**	ATA*GAGCTC*CGCCAACAGTAACGTGT	**690 bp**
**Igf1r Anti-sence**	C*AAGCTT*CCAAACCGAAAACAGGAT	
**Mbnl3 Sence**	ATA*GAGCTC*TAAGCTGGCACTCTAGTTG	**785 bp**
**Mbnl3 Anti-sence**	C*AAGCTT*CTGTAGCTGCTGTTCGTC	

**Table 4:** Sequence of shRNA target to *Myo1d, Camk1d* and *Mbnl3* gene

Gene	Sequence
**iMYO1D-1-F**	**gatcc GCTATTGCTGACGCTGCTTACCGAAGTAAGCAGCGTCAGCAATAGC TTTTTT g**
**iMYO1D-1-R**	**aattc AAAAAA GCTATTGCTGACGCTGCTTACTTCGGTAAGCAGCGTCAGCAATAGC g**
**iMYO1D-2-F**	**gatcc GCAGATGCTGCACTCTCTTCACGAATGAAGAGAGTGCAGCATCTGC TTTTTT g**
**iMYO1D-2-R**	**aattc AAAAAA GCAGATGCTGCACTCTCTTCATTCGTGAAGAGAGTGCAGCATCTGC g**
**Ctrl-iMYO1D-1-F**	**gatcc GATGTCGCAGATGTCGTAGACCGAAGTCTACGACATCTGCGACATC TTTTTT g**
**Ctrl-iMYO1D-1-R**	**aattc AAAAAA GATGTCGCAGATGTCGTAGACTTCGGTCTACGACATCTGCGACATC g**
**iCAMK1D −1-F**	**gatcc GGAAGCTCTTCGCAGTGAAGTCGAAACTTCACTGCGAAGAGCTTCC TTTTTT g**
**iCAMK1D-1-R**	**aattc AAAAAA GGAAGCTCTTCGCAGTGAAGTTTCGACTTCACTGCGAAGAGCTTCC g**
**iCAMK1D-2-F**	**gatcc GCAGCCTGGACAGTTCAAATGCGAACATTTGAACTGTCCAGGCTGC TTTTTT g**
**iCAMK1D-2-R**	**aattc AAAAAA GCAGCCTGGACAGTTCAAATGTTCGCATTTGAACTGTCCAGGCTGC g**
**Ctrl-iCAMK1D-1-F**	**gatcc GATGTCGCAGATGTCGTAGACCGAAGTCTACGACATCTGCGACATC TTTTTT g**
**Ctrl-iCAMK1D-1-R**	**aattc AAAAAA GATGTCGCAGATGTCGTAGACTTCGGTCTACGACATCTGCGACATC g**
**iMBNL3-1-F**	**gatcc GCACTCGTGAGAACTGCAAGTCGAAACTTGCAGTTCTCACGAGTGC TTTTTT g**
**iMBNL3-1-R**	**aattc AAAAAA GCACTCGTGAGAACTGCAAGTTTCGACTTGCAGTTCTCACGAGTGC g**
**iMBNL3-2-F**	**gatcc GCACTTAAAGTCGCAGCTAGACGAATCTAGCTGCGACTTTAAGTGC TTTTTT g**
**iMBNL3-2-R**	**aattc AAAAAA GCACTTAAAGTCGCAGCTAGATTCGTCTAGCTGCGACTTTAAGTGC g**
**Ctrl-iMBNL3-1-F**	**gatcc AGTGCAGCAAGAGTGACTCTCCGAAGAGAGTCACTCTTGCTGCACT TTTTTT g**
**Ctrl-iMBNL3-1-R**	**aattc AAAAAA AGTGCAGCAAGAGTGACTCTCTTCGGAGAGTCACTCTTGCTGCACT g**

Bone marrow-derived dendritic cells (BMDCs) were prepared from the femurs and tibias of sacrificed 4-6 wk old C57BL/6 mice as described [7]. At day 6, the non-adherent, relatively immature DCs were harvested for subsequent assays. 293T cells were cultured in Earle's modified Eagle's medium containing 10% fetal bovine serum (HyClone), 100 units/ml penicillin, and 100 g/ml streptomycin at 37°C under 5% CO_2,_ and used for luciferase reporter assays. Cells were transfected using Lipofectame2000 (Invitrogen).

### MiRNAs selection and quantitative PCR validation

Small RNAs were selected from our previous Microarray data [7]. The complete data set for each miRNA have been listed in supplement1. To test which viral protein mainly charged for the alteration of miRNAs, we amplified four segments (NA, HA, M2 and NS) and then cloned into pcDNA3.1 vector. According to microarray result, 9 up-regulated and 8 down-regulated genes were selected for quantitative PCR (qPCR) verification. Small RNAs were purified using the miRNeasy mini kit (Qiagen), reverse transcribed using the miScript Reverse Transcriptase, and qPCR performed using the QuantiTect SYBR Green PCR master mix (Qiagen). miRNAs expression was normalized to the internal control 5S rRNA. Primers for the 17 selected miRNAs are list in Table 5. All assays were performed in triplicates. Relative expression levels were calculated using the 2-ΔΔCt method [34].

**Table 5:** qRT-PCR primers used for detecting miRNAs alteration

MiRNA	Mibase number	Sence primer
mmu-miR-155-5p	MIMAT0000165	GGGTTAATGCTAATTGTGATAGGGGT
mmu-mir-680-1	MI0004640	CCCGTAGACGACTGTACCCCC
mmu-miR-674-3p	MIMAT0003741	GGGCACAGCTCCCATCTCAGAACA
mmu-miR-222-5p	MIMAT0017061	GGGTCAGTAGCCAGTGTAGATCCT
mmu-miR-221-3p	MIMAT0000669	GGGAGCTACATTGTCTGCTGGG
mmu-miR-707	MIMAT0003497	CAGTCATGCCGCTTGCCTACG
mmu-mir-680-2	MI0004641	CGGGCATCTGCTGACATGGGGG
mmu-miR-22-3p	MIMAT0000531	GGAAGCTGCCAGTTGAAGAACTGT
mmu-miR-499-5p	MIMAT0003482	GGGGTTAAGACTTGCAGTGATGTTT
mmu-miR-375-3p	MIMAT0000739	TTTGTTCGTTCGGCTCGCGT
mmu-miR-29c-3p	MIMAT0000536	GGCGTAGCACCATTTGAAATCG
mmu-miR-146b-5p	MIMAT0003475	GGGGTGAGAACTGAATTCCATAGGCT
mmu-miR-687	MIMAT0003466	GGGCTATCCTGGAATGCAGCAATGA
mmu-miR-24-1-3p	MIMAT0000219	GGTGGCTCAGTTCAGCAGGAAC
mmu-miR-339-5p	MIMAT0000584	TCCCTGTCCTCCAGGAGCTCAC
mmu-miR-181b-5p	MIMAT0000673	AACATTCATTGCTGTCGGTGGGT
mmu-miR-679-3p	MIMAT0017248	GGAGCAAGGTCCTCCTCACAGTAG

### Immune response of BMDCs stimulated by NA

#### Surface marker analysis of BMDCs

Immature BMDCs were plated into fresh medium (1×10^6^ cells/ml) and transfected with constructed vector (NA, HA, M2 and NS), pcDNA3.1 (negative control) and LPS (1 μg/ml, positive control) for 48h. Plasmids were transfected with lipofectame2000 reagent (Invitrogen). Then cells samples (1×10^6^ cells, 1.5 ml tube) were collected, washed twice with PBS and incubated at 4°C for 30 min with the following monoclonal antibodies (anti-mouse CD11c, anti-mouse CD40, anti-mouse CD86, anti-mouse MHC class II and anti-mouse CD80 antibody, respectively). After washing, cells were analyzed with Fluorescence Activated Cell Sorter (FACS) (BD, FACS Aria).

#### Allogeneic mixed leukocytes reaction (MLR) proliferation assays

The primary T-cell stimulatory capacity of BMDCs was examined in a MLR. Untreated and variously treated BMDCs (pcDNA3.1- stimulated, NA-stimulated and LPS-stimulated (100 ng/ml)) were used as the stimulator cells. Allogeneic lymphocytes were obtained from BALB/c as follows. Leukocytes were isolated from the spleens of 4 to 6 week-old mice and cultured in complete RPMI 1640 medium supplemented with 10% FCS in 96-well plates at 37°C for 48 h. Graded numbers of responder cells (1×10^5^ cells/well) were added to 96-well round bottomed plates, giving responder: stimulator ratios of 1:1 or 5:1, in a culture volume of 100 μL. Cell proliferation assays were conducted with the Cell Counting Kit-8 (CCK-8, Beyotime). Each well received 20μL CCK-8 solution and was incubated for a further 2 h at 37°C before absorbance measurement at 450 nm. All experiments were conducted in triplicates. The Stimulation Index was calculated using the formula: SI = (OD_sample_−OD_stimulator cells only_)/(OD_responder cells only_−OD_blank control_) [35].

#### Cytokine measurement

BMDC culture supernatants were collected at 24 h after treatments (Groups were divided as MLR experiments). Concentrations of TNF-α, IL-6, IL-10 and IL-12p70 in the supernatants were measured using the Quantikine Elisa kit (Boster). The sensitivity of the assay was 2 pg/ml for TNF-α, 4 pg/ml for IL-6, IL-10 and IL-12p70.

#### qRT-PCR validation

BMDCs was cultured and collected at 24 h after treatments with NA segment. Then, qPCR was conducted to determine the alteration of Pgm21l, Aldh18a1, Camk1d and Mbnl3, targets of miR674 or miR155. Meantime, we also evaluated the transcription efficient of plasmid pcDNA3.1-NA, M2, NS and HA by qPCR. Primers were all listed in Table 6.

**Table 6:** qRT-PCR primers used for detecting target genes and viral segments

Gene name	Sence primer	Anti-Sence primer
Pgm2l1	cctccaattccagtcccaga	atccaccacccaaacaaagc
Aldh18a1	tgtaatgccctggagacgtt	gacttcacttctgaggggct
Camk1d	tccgactctgccaaagactt	acgcttgtctccatttgctc
Mbnl3	gctatgctcaccctacggat	gatgagctgccctgagtttg
MYO1D	attcgaacaccccgtacact	ttggccacttcacatgcttc
NA	GCAGAGACAATTGGAAGGGC	CATTCCCATCGTCAAAGGCC
HA	TGGGAAGGGATGCTTCGAAT	CATGGCCCAGAACATGAAGG
NS	TCCTTCCCGAGTAGCAGTTC	ACTGTGTCAAGCTTCCAGGT
M2	AGATGGCGACTACCACCAAC	AGTCCCAATTGTCCTCATCG

### Immune response of BMDCs stimulated by miRNAs

#### Plasmid construction and phenotypic detection

To confirm the phenotype alteration induced by NA segment may meditated by miRNAs. MiRNAs over-expression vector were constructed based on pSilencer4.1 vector (Invitrogen). Four selected miRNAs (miR155, miR499, miR674 and miR181b1) were amplified and then cloned into pSilencer4.1, whose primers were listed in Table 2. The isolation of BMDCs and phenotypic detection were as previous description.

#### MLR proliferation assays, Cytokines analysis and qRT-PCR validation

Cells were grouped as non-stimulated, pSilencer4.1-stimulated (negative control), miR155- or miR674-stimulated groups and LPS-stimulated (positive control). MLR detection was performed as previous. Moreover, cells culture supernatants were collected at 24 h for cytokines detection. The concentrations of TNF-α, IL-6, IL-10 and IL-12p70 were measured as previous. Furthermore, BMDCs was cultured and collected at 24 h after treatments with miR674 or miR155. Then, qPCR was conducted to determine the alteration of Pgm21l, Aldh18a1, Camk1d and Mbnl3, targets of miR674 or miR155.

### MiRNAs inhibiting experiment

To detect whether the phenotypic alteration of BMDCs induced by NA was mediated by miRNAs, miRNAs inhibitors were designed and purchased from RIBBIO (Guangzhou, China). Each 100 nM miRNAs inhibitors (miR155, miR499, miR674 and miR181b1) were transfected into BMDCs for 2h, before NA over-expression plasmid was transfected. After another 24 h, BMDCs were collected for phenotypic detection with FACS.

### MiRNA target prediction and validation

Target genes of miR-155 and miR-674 were predicted using miRanda and Targetscan [36]. Only target genes identified by these two algorithms were considered. Besides, we matched predicted genes with our previous microarray data to narrow the target. The 3′-UTRs of selected target genes *(Myo1d, Pgm2l1, Aldh18a1, Entpd6, Camk1d, Igf1r* and *Mbnl3)*, harboring the predicted miR155 and miR674 binding sequences, were amplified and cloned into pMIR-Report luciferase vector (Ambion, TX) (Table 3). Meantime, the mutated vector was constructed by overlap PCR methods. The predicted genes target position and mutated position of miR155 and miR674 was listed in Table 7. To determine whether miR-155 and miR-674 could repress the expression of target genes, 293T cells were transfected with miR-155 or miR-674 and pMIR-Report vector, along with pRL-TK to normalize transfection efficiencies. Luciferase assays were performed using the dual-luciferase reporter assay system kit (Promega), according to the manufacturer's protocol, on a Modulus single-tube multimode reader (Promega).

**Table 7:** The predicted genes target position and mutated position of miR155 and miR674

Gene	Sequence	Position
**Myo1d-mut**	5′…CAUCAAGAGCAAUAAATCTAGCAA…	
**Myo1d 3′ UTR**	5′…CAUCAAGAGCAAUAAAGCAUUAA…	**1922-1929**
**mmu-miR-155**	3′…UGGGGAUAGUGUUAAUCGUAAUU…	
**Pgm2L1-mut**	5′…AUUCUUGUCAAAUUCCTCTAGGC…	
**Pgm2L1 3′ UTR**	5′…AUUCUUGUCAAAUUCCUCAGUGC…	**618-624**
**mmu-miR-674**	3′…AUGUGGUGAGGGUAGAGUCACG…	
**Entpd6-mut**	5′…GCUUCCCCAUGGCCCCTCTAGGA…	
**Entpd6 3′ UTR**	5′…GCUUCCCCAUGGCCCCUCAGUGA…	**848-855**
**mmu-miR-674**	3′…AUGUGGUGAGGGUAGAGUCACG	
**Aldh18a1-mut**	5′…UGGCUGCCGGAACGCCTCTAGGA…	
**Aldh18a1 3′ UTR**	5′…UGGCUGCCGGAACGCCUCAGUGA…	**416-423**
**mmu-miR-674**	3′…AUGUGGUGAGGGUAGAGUCACG…	
**Camk1d -mut**	5′…CACCACUUCCGCUCUCTCTAGGU…	
**Camk1d 3′ UTR**	5′…CACCACUUCCGCUCUCUCAGUGU…	**305-311**
**mmu-miR-674**	3′…AUGUGGUGAGGGUA—GAGUCACG…	
**IGF1R -mut**	5′…ACAAGCCUCCUGUACCTCTAGGG…	
**IGF1R 3′ UTR**	5′…ACAAGCCUCCUGUACCUCAGUGG…	**115-121**
**mmu-miR-674**	3′…AUGUGGUGAGGGUA–GAGUCACG…	
**Mbnl3 -mut**	5′…AUCUUCCUAAAGAGGCTCTAGGG…	
**Mbnl3 3′ UTR**	5′…AUCUUCCUAAAGAGGCUCAGUGG…	**9327-9333**
**mmu-miR-674**	3′…AUGUGGUGAGGGUAGAGUCACG…	

### Phenotypic analysis of target genes shRNA-transfected DCs

#### Construction of recombinant lentivirus vectors

To confirm whether miR-155 and miR-674 activated the immune function of DCs by inhibiting their target genes. The knockdown of *Myo1d, Camk1d* and *Mbnl3* in BMDCs was performed by RNA interference (RNAi) based on vector pLVX-shRNA. The targeting sequence of shRNA against Myo1d, Camk1d and Mbnl3 were designed using online design tools (http://rnaidesigner.lifetechnologies.com/rnaiexpress/design.do; Block-iT RNAi Designer). Six pairs of siRNA oligonucleotides targeting 3 target genes were designed and synthesized by Invitrogen, which were listed in Table 4. The annealed oligonucleotides were cloned into the BamHI and EcoRI sites of pLVX-shRNA1 using compatible restriction sites flanking the oligo siRNA target sequence. We also generated a mutated siRNA control construct for each target gene, which is not predicted to target any genes (Table 4). All clones were verified by DNA sequencing.

#### Lentivirus packaging

The pseudotyped lentiviruses were produced in 293T cells by co-transfection with the recombinant siRNA plasmids (siMyo1d-1, siMyo1d-3, Ctrl-siMyo1d-5, siCamk1d-7, siCamk1d-9, Ctrl-siCamk1d-11, siMbnl-13, siMbnl-15 and Ctrl-siMbnl-17) and the three Clontech Lenti-X HT packaging vectors. After 48 h of transfection, the cell supernatants were collected and centrifuged at 500×g for 10 min; the soluble supernatant fractions containing the lentiviruses were collected and stored for later using.

#### Phenotypic analysis of shRNA-transfected DCs

BMDCs were transfected with siMyo1d, siCamk1d, siMbnL3 and their Ctrl-siRNAs at 37° in 5% CO2 for 48 h. Then, BMDCs were collected to detect the RNA interference efficiency by qPCR. Then BMDCs were infected with the effective interference plasmid and part of cells were subsequently stained with phycoerythrin- or fluorescein isothiocyanate-conjugated monoclonal antibodies (mAbs) against mouse CD40, CD80, CD86 and mouse MHC class II molecules for 30 min at 4°C. Negative controls were isotype-matched mAbs. After washing, the cells were suspended and examined by FACS. Furthermore, the other part of DCs was transfected with miR155 or miR674 after target genes were silenced for 24 h. Finally, this part of BMDCs was stained and examined by FACS.

### Western blot assay

Since DCs were the only bridge communicated the innate and acquired immunity, we tried to evaluated how NA segment and their induced miRNAs affect the TLR and NF-kB signal pathways by western blot. BMDCs were transfect with NA and miR155 or miR674 over expression plasmid for 24h. Then cells were collected and washed with PBS three times for the next experiments. Western blot detection was performed as our previous article [7]. Mouse IkBa, P-IkBa, P38, P-P38, ERK, P-ERK, JUK, P-JUK, IRF-3, IRF-7, TRAF-3 and TRAF-6 were selected and detected according to each manufacturer's protocol. Protein bands were visualized using the Super ECL Plus system. GAPDH was used as a loading control.

### Virus replication experiments

DCs were seeded onto 6-well plates (5 × 10^5^ cells /well) and pretreated with miRNAs inhibition or over-expression plasmid for 24 h. Then, pretreated DCs were inoculated with 100 μL H9N2 AIV for 1 h at 37 °C (Virus was diluted in PBS with 10^6^ TCID_50_/100μL). After that, DCs were washed five times with 1000 rmp/5 min to eliminate the non-absorbed virus and resuspended in fresh RPMI 1640 medium. After another 24 h, BMDCs were collected and total RNA was extracted with trizol reagents. RT-PCR was then performed according to instructions (TaKaRa). For real-time PCR, 7500 Real-Time PCR System (ABI) and SYBR Green Master (Takara) were used. Virus PB1 gene was choose as the target gene and the mouse GAPDH gene was used as the internal parameter (primers were list in Table.8). Quantification of the target gene was determined by relative standard curves. PB1 and GAPDH plasmids were used to produce a double-standard curve. The mouse GAPDH quantity at each sampling time was taken as the standard to determine the target genes quantity.

**Table 8:** Primers used in the absolute q RT-PCR for detecting virus replication

Gene	Sequence	Products
**PB1 Sence**	AGCGGGTATGCACAAACAGA	**150 bp**
**PB1 Anti-sence**	ATAAGTCTGGCGACCTTGGG	
**GAPDH Sence**	AACTTTGGCATTGTGGAAGG	**223 bp**
**GAPDH Anti-sence**	ACACATTGGGGGTAGGAACA	

**Table 9:** Primers used in amplifying Myo1d, Caml1d and Mbnl3

Gene	Sequence		Products
**Myo1d Forword**		GGGTACC**atggcggagcaggagagcc**	**3034bp**
**Myo1d reverse**		GCTCGAG**tcagttcccgggcacactg**	
**Camk1d Forword**		GGGTACC**atggcccgggagaacggc**	**1160 bp** **1035bp**
**Camk1d reverse**		GCTCGAG**tcacttgcttccagtgtgcc**	
**Mbnl3 Forword**		GGGTACC**atgacacctgtcaatgtagc**	
**Mbnl3 reverse**		GGGTACC**tcaatatttcaactggttgcctg**	

### Statistical analysis

Data were evaluated by unpaired two-tailed Student's t-test using GraphPad Prism 5 (http://www.graphpad.com) (CSSN), with p values < 0.05 considered to be statistically significant. The significance of the data was also determined by one-way ANOVA, followed by Tukey's multiple comparison tests. FACS data were analysed by FlowJo software (FlowJo, China). All data are expressed as mean ± standard error of the mean.

## SUPPLEMENTARY MATERIALS FIGURES AND TABLE




