# Supplementary material for: MiR674 inhibits the neuraminidase-stimulated immune response on dendritic cells via down-regulated Mbnl3
Source: Oncotarget. 2016 Jun 6;7(31):48978–94. doi: 10.18632/oncotarget.9832 (PMC5226485; doi:10.18632/oncotarget.9832)
Supplement: Supplementary file 1 [file oncotarget-07-48978-s001.pdf]

MiR674 inhibits the neuraminidase-stimulated immune response on dendritic cells via down-regulated Mbnl3

Supplementary Material

Supplement.1: Selected data of alteration miRNAs stimulated by H9N2 AIV

Gene Symbol	RefSeq	Fold-Change	
		H9N2 vs. Control	Inactivated H9N2 vs. control
miR155	NR_029565	4.4388	3.37428
miR680-1	NR_030447	2.78118	1.65605
miR674	NR_030440	2.66297	1.95109
miR222	NR_029807	1.92629	2.67773
miR221	NR_029806	1.83013	1.82881
miR707	NR_030488	1.74317	1.21702
miR680-2	NR_030448	1.66477	1.13492
miR22	NR_029739	1.54757	1.27699
miR499	NR_030757	1.48486	1.76494
miR375	NR_029876	-1.37088	-1.58239
miR29c	NR_029745	-1.40399	-1.62141
miR146b	NR_030468	-1.44245	-1.3809
miR687	NR_030459	-1.56986	-1.66524
miR24-1	NR_029575	-1.59192	-1.66838
miR339	NR_029768	-1.71149	-1.97229
miR181b-1	NR_029820	-3.05312	-3.04058
miR679	NR_030445	-1.26432	-1.66074

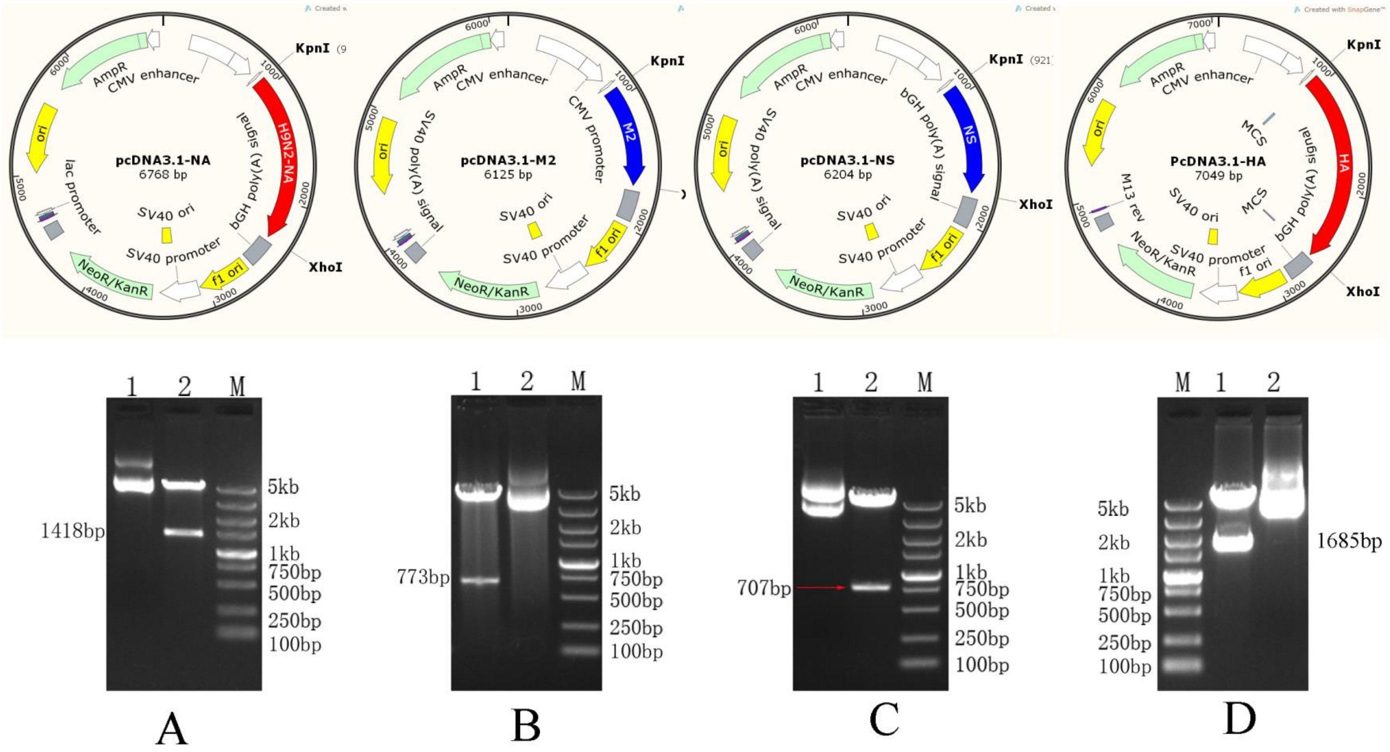

Supplement.2： Identification and construction of pcDNA3.1-NA, pcDNA3.1-M2, pcDNA3.1-NS and pcDNA3.1-HA
(A: Identification of pcDNA3.1-NA by digesting with Xho I and Kpn I. M: DL5000 DNA Marker, 1: Plasmid pcDNA3.1-NA,
2: Plasmid pcDNA3.1-NA digested with Xho I and Kpn I;
B:Identification of pcDNA3.1-M2 by digesting with Xho I and Hind III;
C: Identification of pcDNA3.1-NS by digesting with Xho I and Kpn I ;
D: Identification of pcDNA3.1-HA by digesting with Xho I and Kpn I).

Supplement 2. Construction and identification of plasmid pcDNA3.1-NA, pcDNA3.1-M2, pcDNA3.1-NS and pcDNA3.1-HA

A: Identification of pcDNA3.1-NA by digestion with *Xho*I and *Kpn*I (M1: DL5000 DNA marker; 1: plasmid pcDNA3.1-NA; 2: plasmid pcDNA3.1-NA digested with *Xho*I and *Kpn*I).

B: Identification of pcDNA3.1-M2 by digestion with *Xho*I and *Hin*dIII (M1: DL5000 DNA marker; 1: plasmid pcDNA3.1-M2; 2: plasmid pcDNA3.1-M2 digested with *Xho*I and *Hin*dIII).

C: Identification of pcDNA3.1-NS by digestion with *Xho*I and *Kpn*I (M1: DL5000 DNA marker; 1: plasmid pcDNA3.1-NS; 2: plasmid pcDNA3.1-NS digested with *Xho*I and *Kpn*I).

D: Identification of pcDNA3.1-HA by digestion with *Xho*I and *Kpn*I (M1: DL5000 DNA marker; 1: plasmid pcDNA3.1-HA; 2: plasmid pcDNA3.1-HA digested with *Xho*I and *Kpn*I).

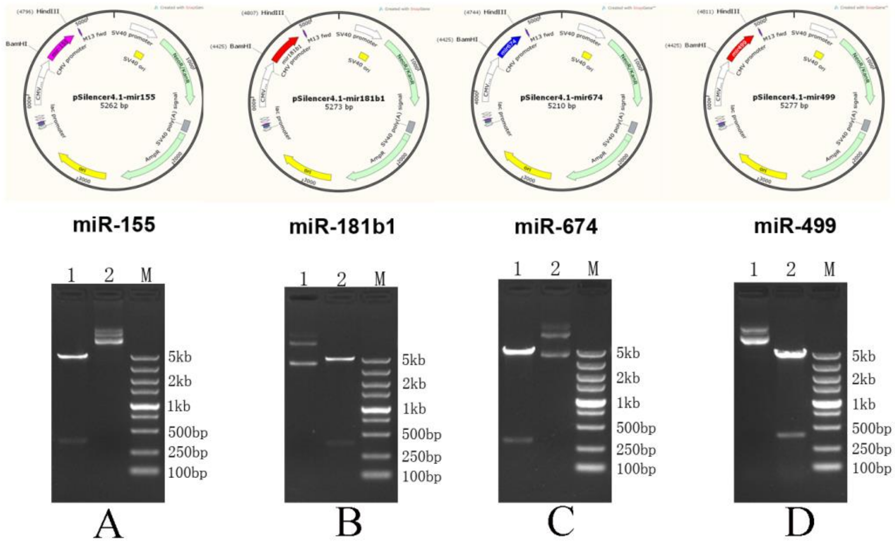

Supplement.3： Identification and construction of pSilencer-miR155, pSilencer-miR181b1, pSilencer-miR674 and pSilencer-miR499 .

(A: Identification of pSilencer-miR155 by digesting with BamH I and Hind III. M1: DL5000 DNA Marker,

1: Plasmid pSilencer-miR155 digested with BamH I and Hind III； 2: Plasmid pSilencer-miR155;

B:Identification of pSilencer-miR181b1 by digesting with BamH I and Hind III.;

C: Identification of pSilencer-miR674 by digesting with BamH I and Hind III;

D:Identification of pSilencer-miR499 by digesting with BamH I and Hind III.).

Supplement 3. Construction and identification of plasmid pSilencer-miR155, pSilencer-miR181b1, pSilencer-miR674, and pSilencer-miR499

A: Identification of pSilencer-miR155 by digestion with *Bam*HI and *Hin*dIII (M1: DL5000 DNA marker; 1: pSilencer-miR155 digested with *Bam*HI and *Hin*dIII; 2: plasmid pSilencer-miR155).

B: Identification of pSilencer-miR181b1 by digestion with *Bam*HI and *Hin*dIII (M1: DL5000 DNA marker; 1: plasmid pSilencer-miR181b1; 2: pSilencer-miR181b1 digested with *Bam*HI and *Hin*dIII).

C: Identification of pSilencer-miR674 by digestion with *Bam*HI and *Hin*dIII (M1: DL5000 DNA marker; 1: pSilencer-miR674 digested with *Bam*HI and *Hin*dIII; 2: plasmid pSilencer-miR674).

D: Identification of pSilencer-miR499 by digestion with *Bam*HI and *Hin*dIII (M1: DL5000 DNA marker; 1: plasmid pSilencer-miR499; 2: pSilencer-miR499 digested with *Bam*HI and *Hin*dIII).

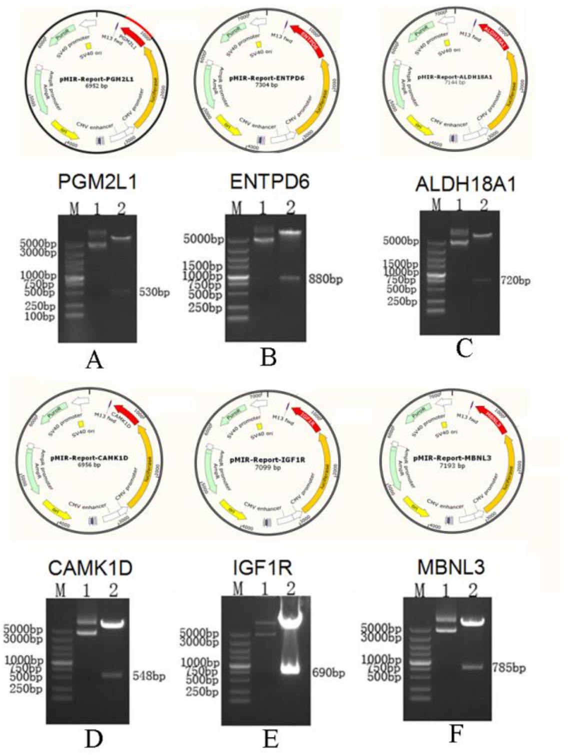

Supplement 4: Identification and construction of pMIR-REPORT-Pgm2l1, Entpd6, Aldh18a1, Camk1d, Igf1r and Mbnl3..

(A: Identification of pMIR-REPORT-Pgm2l1 by digesting with Sac I and Hind III; M1: DL5000 DNA Marker, 1: Plasmid pMIR-REPORT-Pgm2l1, 2: Plasmid pMIR-REPORT-Pgm2l1 digested with Sac I and Hind III; B-F:Identification of constructed pMIR-REPORT plasmid by digesting with Sac I and Hind III; M1: DL5000 DNA Marker, 1: Plasmid pMIR-REPORT, 2: Plasmid pMIR-REPORT digested with Sac I and Hind III).

Supplement 4. Construction and identification of plasmid pMIR-REPORT-Pgm2l1, Entpd6, Aldh18a1, Camk1d, Igf1r and Mbnl3

A: Identification of pMIR-REPORT-Pgm2l1 by digesting with Sac I and Hind III;

M1: DL5000 DNA Marker, 1: Plasmid pMIR-REPORT-Pgm2l1,

2: Plasmid pMIR-REPORT-Pgm2l1 digested with Sac I and Hind III;

B-F: Identification of constructed pMIR-REPORT plasmid by digesting with Sac I and Hind III; M1: DL5000 DNA Marker, 1: Plasmid pMIR-REPORT, 2: Plasmid pMIR-REPORT digested with Sac I and Hind III).

Supplement 5. Results of the qPCR analysis of the transfection efficient on BMDCs with plasmid pcDNA3.1-NA, pcDNA3.1-M2, pcDNA3.1-NS and pcDNA3.1-HA

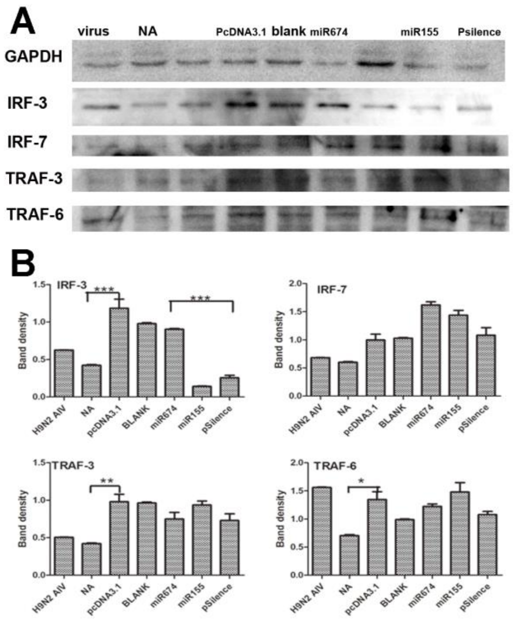

Supplement 6. Regulatory protein expression on BMDCs stimulated by NA, miR-155 and miR-674 as determined by Western blotting

A: Western blot results for IRF-3, IRF-7, TRAF-3, and TRAF-6 in cells stimulated by NA, miR-155 and miR-674 (Lanes 1: H9N2 stimulated group; lane 2: NA stimulated group; lane 3: pcDNA3.1 stimulated group; Lanes 4: Blank group; Lanes 5: miR-674 stimulated group; lane 6: miR-155 stimulated group; lane 7: pSilencer4.1 stimulated group).

B: The expression levels of IRF-3, IRF-7, TRAF-3, and TRAF-6 in cells stimulated by NA, miR-155 and miR-674 (the data shown are the means ± standard error of the mean of double wells from three independent experiments). The levels of significance are identified by $P<0.05$ or $P<0.01$.

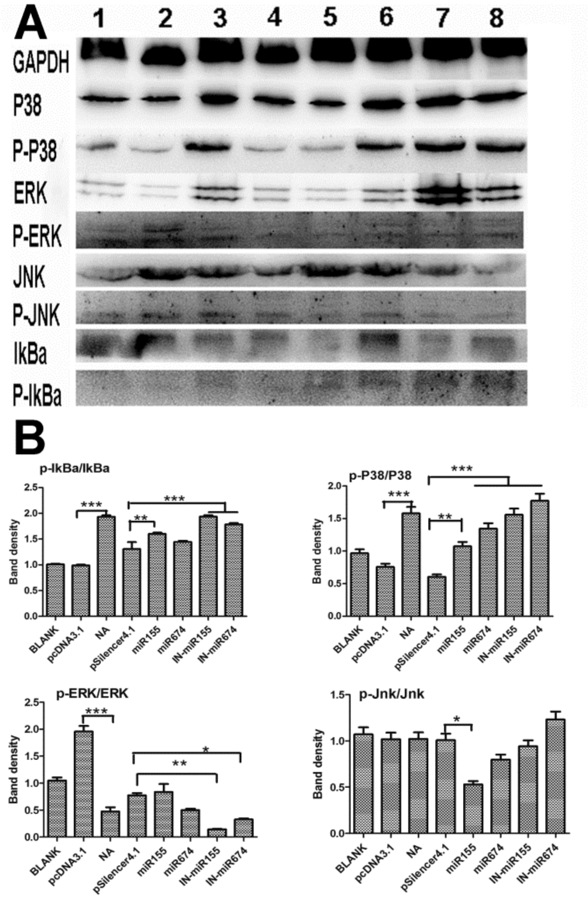

Supplement 7. Regulatory protein expression of MAPK signal pathway on BMDCs stimulated by NA, miR-155 and miR-674 as determined by Western blotting

A: Western blot results for IkBa, P38, ERK and Jnk in BMDCs stimulated by NA, miR-155, miR-674, IN-miR155 and IN-miR674 (Lanes 1: Blank group; lane 2: pcDNA3.1 stimulated group; lane 3: NA stimulated group; Lanes 4: pSilencer4.1 stimulated group; Lanes 5: miR-155 stimulated group; lane 6: miR-674 stimulated group; lane 7: miR-155 inhibited group; lane 8: miR-674 inhibited group).

B: The expression levels of IkBa, P38, ERK and Jnk in BMDCs stimulated by NA, miR-155, miR-674, IN-miR155 and IN-miR674 (the data shown are the means ± standard error of the mean of double wells from three independent experiments). The levels of significance are identified by *P<0.05* or *P<0.01*.

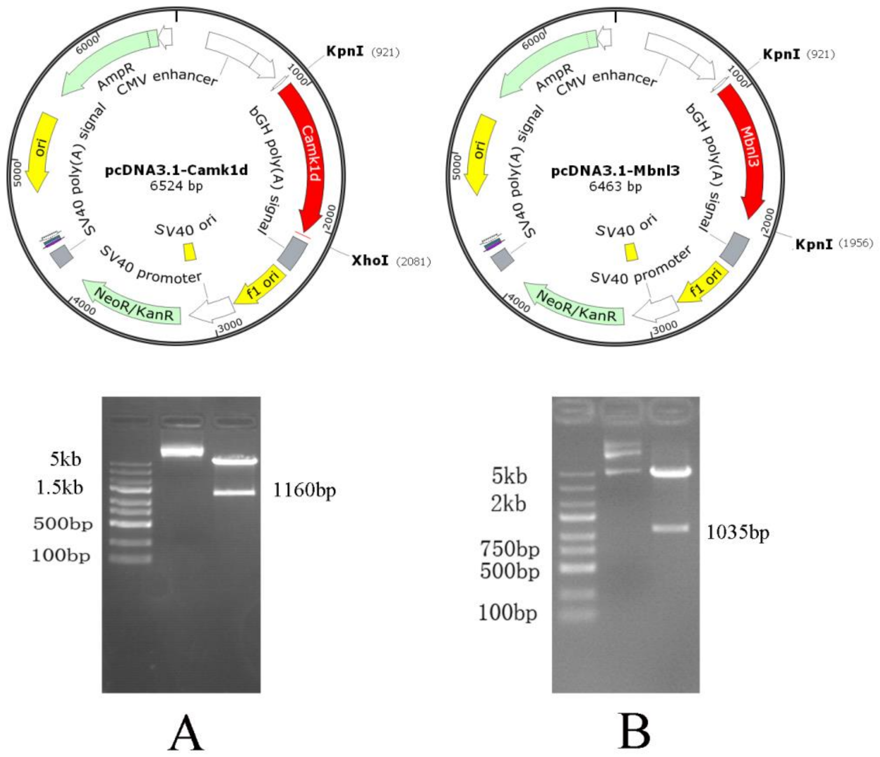

Supplement. : Identification and construction of pcDNA3.1-Camk1d and pcDNA3.1-Mbnl3

A: Identification of pcDNA3.1-Camk1d by digesting with Xho I and Kpn I. ;

B:Identification of pcDNA3.1-Mbnl3 by digesting with Kpn I.

Supplement 8. Construction and identification of plasmid pcDNA3.1-Camk1d and pcDNA3.1-Mbnl3

A: Identification of pcDNA3.1-Camk1d by digestion with *Xho*I and *Kpn*I (M1: DL5000 DNA marker; 1: plasmid pcDNA3.1-Camk1d; 2: plasmid pcDNA3.1- Camk1d digested with *Xho*I and *Kpn*I).

B: Identification of pcDNA3.1-Mbnl3 by digestion with *Kpn*I (M1: DL5000 DNA marker; 1: plasmid pcDNA3.1- Mbnl3; 2: plasmid pcDNA3.1- Mbnl3 digested with *Kpn*I).

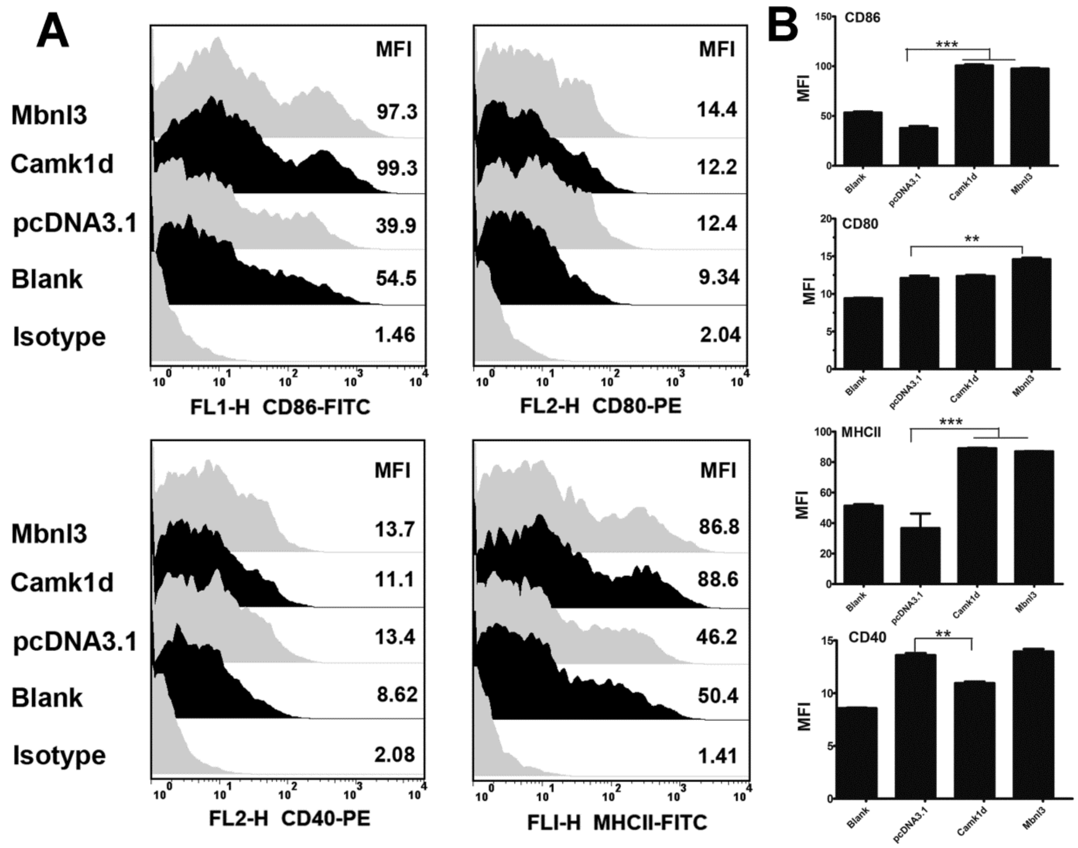

Supplement 9. Immune activation of BMDCs stimulated by pcDNA3.1-Camk1d and pcDNA3.1-Mbnl3

A: Flow cytometric analysis of the phenotypic alterations in DCs stimulated with pcDNA3.1-Camk1d and pcDNA3.1-Mbnl3 (i.e., the expressions of CD40, CD80/86, and MHCII on BMDCs stimulated with pcDNA3.1-Camk1d and pcDNA3.1-Mbnl3).

B: The MFI of CD40, CD80/86, and MHCII (Significant differences between the treated and control groups are expressed as **P<0.05* or ***P<0.01)*.

Supplement 5. Results of the qPCR analysis of the transfection efficient on BMDCs with plasmid pcDNA3.1-NA, pcDNA3.1-M2, pcDNA3.1-NS and pcDNA3.1-HA.

For Table S5, please see the attached Excel file
